# pH-Responsive Aqueous Bubbles Stabilized With Polymer Particles Carrying Poly(4-vinylpyridine) Colloidal Stabilizer

**DOI:** 10.3389/fchem.2018.00269

**Published:** 2018-07-17

**Authors:** Masaya Ito, Koki Takano, Haruka Hanochi, Yuta Asaumi, Shin-ichi Yusa, Yoshinobu Nakamura, Syuji Fujii

**Affiliations:** ^1^Graduate Course in Applied Chemistry, Environmental and Biomedical Engineering, Graduate School of Engineering, Osaka Institute of Technology, Osaka, Japan; ^2^Department of Applied Chemistry, Faculty of Engineering, Osaka Institute of Technology, Osaka, Japan; ^3^Department of Applied Chemistry, Graduate School of Engineering, University of Hyogo, Hyogo, Japan; ^4^Nanomaterials Microdevices Research Center, Osaka Institute of Technology, Osaka, Japan

**Keywords:** bubbles, stimulus-responsive, polymer, particles, interface, adsorption

## Abstract

Free radical dispersion polymerization was conducted to synthesize near-monodispersed, micrometer-sized polystyrene (PS) particles carrying pH-responsive poly(4-vinylpyridine) (P4VP) colloidal stabilizer (P4VP-PS particles). The P4VP-PS particles were extensively characterized in terms of morphology, size, size distribution, chemical composition, surface chemistry, and pH-response using optical and scanning electron microscopies, elemental microanalysis, X-ray photoelectron spectroscopy, laser diffraction particle size analysis, and zeta potential measurement. The P4VP-PS particles can work as a pH-responsive stabilizer of aqueous bubbles by adsorption at the air-water interface. At and above pH 4.0, where the particles have partially protonated/non-protonated P4VP stabilizer with relatively hydrophobic character, particle-stabilized bubbles were formed. Optical and scanning electron microscopy studies confirmed that the P4VP-PS particles were adsorbed at the air-water interface of the bubbles in aqueous media. At and below pH 3.0, where the particles have cationic P4VP stabilizer with water-soluble character, no bubble was formed. Rapid disruption of the bubbles can be induced by decreasing the pH; the addition of acid caused the *in situ* protonation of pyridine groups in P4VP, which impart water-soluble character to the P4VP stabilizer, and the P4VP-PS particles were desorbed from the air-water interface. The bubble stabilization/destabilization cycles could be repeated at least five times.

## Introduction

For a long time, it has been known that gas bubbles can be stabilized solely by solid colloidal particles in aqueous media (Ramsden, 1903; Binks and Horozov, 2006; Studart et al., 2006; Fujii and Murakami, 2008; Hunter et al., 2008; Kruglyakov et al., 2011; Stevenson, 2012; Pugh, 2016; Fujii and Nakamura, 2017). The hydrophilicity-hydrophobicity balance of the particle surfaces determines the adsorption behavior of such particles at the air-water interface, and the bubbles stabilized by solid particles of the suitable hydrophilicity-hydrophobicity balance show excellent long-term stability. Disruption of bubbles is also often required in practical applications. It has been shown that the stability of bubbles stabilized with stimulus-responsive solid particles can be controlled *in situ* by application of external stimulus such as pH, temperature, light and magnetic fields, as reviewed recently (Fujii and Nakamura, 2017). In such cases, disruption of bubbles can be realized by decreasing the adsorption energy of the solid particles at the interface or by application of external energy exceeding the adsorption energy.

Recently, we synthesized polystyrene (PS) particles carrying poly[2-(diethylamino)ethyl methacrylate] (PDEA) colloidal stabilizer (PDEA-PS particles), and evaluated their ability as a pH-responsive particulate bubble stabilizer (Fujii et al., 2011; Nakayama et al., 2015b, 2016). Using this system, bubbles stabilized under basic conditions (>pH 7) can be destabilized by the addition of an acidic solution. This addition leads to protonation of the PDEA on the PS particle surfaces, which makes the PDEA hydrophilic, and afterward the PDEA-PS particles are desorbed from the air-water interface, leading to disruption of the bubbles. The critical maximum pH required for destabilization of the bubbles correlates closely with the p*K*_a_ value of 7.6 for PDEA chains. These results indicated that the threshold pH value which determines bubble stability depends on the pH-responsive nature (p*K*_a_ value) of the colloidal stabilizer on the particle surfaces. We also demonstrated that PS particles carrying poly[2-(dimethylamino)ethyl methacrylate] (PDMA) colloidal stabilizer can work as the pH-responsive particulate bubble stabilizer. The p*K*_a_ value of PDMA is 7.0 and the threshold pH value determining the bubble stability was near pH 7 (Fujii et al., 2015). In order to widen application ranges of the stimulus-responsive particle-stabilized bubbles, it is crucial to control the threshold pH value which determines their stability.

**Figure 1:**
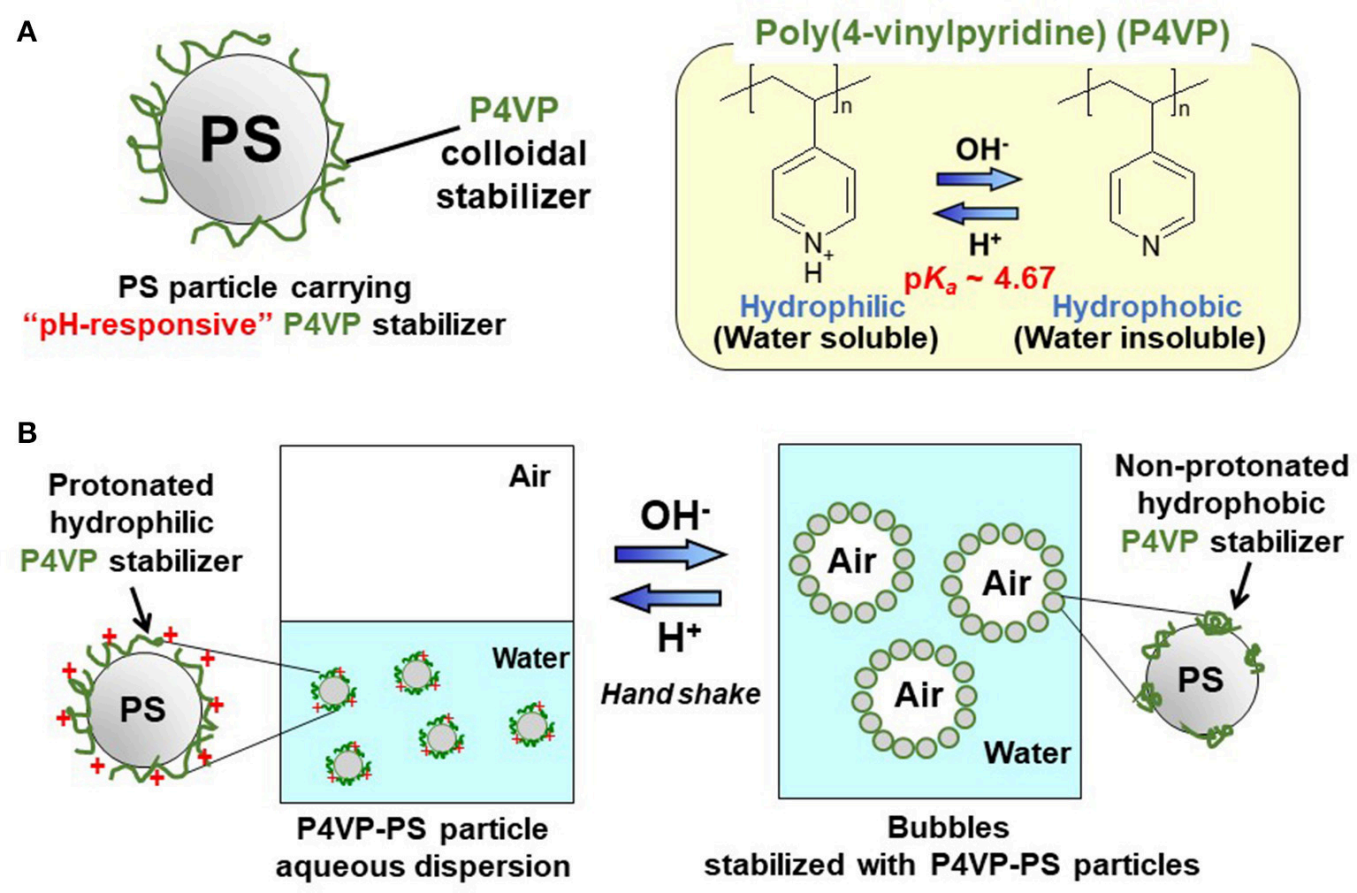
**(A)** Diagram of polystyrene particle carrying pH-responsive poly(4-vinylpyridine) colloidal stabilizer (P4VP-PS particle) and **(B)** schematic diagram illustrating pH-responsive bubbles stabilized with the P4VP-PS particles.

In the present study, there are two objectives: (i) synthesis and characterization of near-monodispersed, micrometer-sized PS particles carrying poly(4-vinylpyridine) (P4VP) colloidal stabilizer on their surfaces (P4VP-PS particles) by free radical dispersion polymerization, and (ii) investigation of their ability as a pH-responsive particulate bubble stabilizer (Figure 1). P4VP shows a p*K*_a_ value of ~4.5 (Wang et al., 2017), which is lower than those of PDEA and PDMA, and it is expected that stability of aqueous bubbles stabilized with the P4VP-PS particles should be changed at around pH 4.5. The P4VP-PS particles were characterized in terms of morphology, size, size distribution, chemical composition, surface chemistry, and pH-response. The particle-stabilized bubbles were characterized in detail with respect to their stability, microstructure and pH response.

## Experimental

### Materials

Styrene, α,α′-azobisisobutyronitrile (AIBN), isopropanol (IPA, 99%), hydrochloric acid (HCl, 0.5 M aqueous solution), sodium hydroxide (NaOH, ≥ 98%), 4-vinylpyridine (4VP, 95%), PS homopolymer (M.W. 45,000), and aluminum oxide (activated, basic, Brockmann 1, standard grade) were purchased from Sigma-Aldrich. Ethyl 2-cyanoacrylate (Aron Alpha Extra Sokkotayoto) was purchased from Toagosei Co. The inhibitors in styrene and 4VP monomers were removed by treatment with the basic alumina. Water was first ion exchanged and then distilled (Advantec MFS RFD240NA: GA25A-0715).

### Preparation of P4VP homopolymer by solution polymerization

Solution of IPA (100 mL) and the free radical initiator AIBN (0.1 g, 0.61 mmol) was prepared in a 250 mL flask and gas phase was replaced with nitrogen gas to purge oxygen at room temperature. Then, the monomer 4VP (10.0 g, 95 mmol) was introduced to the flask in order to start the free radical solution polymerization using a temperature-controlled magnetic stirrer with constant stirring at 250 rpm at 70°C. The resulting solution was cooled to room temperature after the polymerization for 24 h.

### Preparation of P4VP-stabilized PS (P4VP-PS) particles by dispersion polymerization

The IPA solution of P4VP homopolymer prepared by the free radical solution polymerization (4.55 g, 10.24 wt%), the initiator AIBN (50 mg, 0.30 mmol) and ethanol (44.5 mL) were mixed in a 250 mL flask equipped with a magnetic stirrer bar, and gas phase was replaced with nitrogen gas. The polymerization was started by injection of styrene (5.0 g, 48.0 mmol) to the flask at 70°C with constant stirring at 250 rpm. After 24 h, P4VP-PS latex was cooled down to 25°C to stop the polymerization. Purification of the latex was conducted by centrifugation/redispersion cycles with ethanol (3 cycles) and then deionized water (5 cycles) using a centrifuge.

### Characterization of P4VP-PS particles and bubbles

#### Optical microscopy (OM)

A drop of an aqueous dispersion of the P4VP-PS particles or aqueous bubbles was placed on a microscope slide glass and observed using an OM (Shimadzu Motic BA200; Shimadzu Corp., Kyoto, Japan) fitted with an objective lens and a digital system (Shimadzu Moticam, 2000). For observation of the P4VP-PS particles, a cover glass was placed on the sample.

#### Scanning electron microscopy (SEM)

The purified latex droplets dried on an aluminum stub were sputter-coated with thin layer of Au using an Au coater. SEM studies were conducted using a Keyence VE-8800 SEM operated at 5 kV. Number-average diameter of the P4VP-PS particles was evaluated from the SEM images. Dried bubbles were also observed using SEM.

#### Particle size analysis

A laser diffraction particle size analyzer (Malvern Mastersizer, 2000), which is equipped with a small volume sample dispersion unit, a solid-state blue laser (466 nm) and a HeNe laser (633 nm), was utilized to determine volume equivalent sphere mean diameter (*D*_v_). The resulting data are presented as mean diameter ± standard deviation.

#### Chemical composition

The P4VP loading of the P4VP-PS particles after washing was determined by comparing the nitrogen contents evaluated by elemental microanalysis (Yanaco CHN-Corder MT-5) with that of the P4VP homopolymer.

#### X-ray photoelectron spectroscopy (XPS)

The XPS measurements were obtained on the samples mounted onto sample stubs using conductive tape using an XPS spectrometer (Axis Ultra) with a monochromated Al K_α_ X-ray gun.

#### Zeta potential

The electrophoretic mobilities were measured to determine zeta potentials using a Malvern Zetasizer Nano ZS. Measurements were carried out as a function of pH with diluted latex by a gradual addition of HCl or NaOH starting from an initial pH of 6.9.

### Interfacial particle trapping method

Particle trapping at air-water interface was carried out following the method reported in 2014. (Vogel et al., 2014). Aqueous dispersion of P4VP-PS particles (pH 10.0 adjusted using NaOH aqueous solution) placed in a petri dish was magnetically stirred at 450 rpm for 10 min. The ethyl 2-cyanoacrylate monomer (0.7 g) is placed in the other petri dish on a hotplate (50°C). Both petri dishes were placed in a closed glass container for 15 min. The monomer can evaporate and polymerize at the air–water interface. The anionic polymerization of cyanoacrylate occurs by contact with water surface, resulting in generation of the polycyanoacrylate film at the air-water interface. The P4VP-PS particles were trapped at the air-water interface in their equilibrium position.

### Bubble preparation

The pH of the original dispersion after centrifugal washing was 6.9; the pH was controlled by the addition of concentrated aqueous solutions of either NaOH or HCl. The aqueous latex (2.0 mL, solid content, 5.0 wt%) prepared in a glass vessel (4 mL) with a screw cap was hand shaken for 30 s (70 cycles; amplitude of a swing, 30 cm). The same experiments were also conducted using 5.0 mL aqueous latex in a glass vessel (13.5 mL). Prepared bubbles were stored at 25°C and their heights were measured using a ruler. (The bubble height was determined to be 0 mm, if the planar air-aqueous latex interface could be observed).

## Results and discussion

P4VP is a pH-responsive polybase with a p*K*_a_ value of ~4.5 (Wang et al., 2017), which is lower than those of PDEA and PDMA. Relationship between degree of protonation of the pyridine group (α) and pH can be expressed using Equation (1)

(1) $$\begin{array}{r} \alpha =1/\left(1+10^{\text{pH}-\text{p}\text{K}a}\right) \end{array}$$

where *K*_a_ is proton dissociation constant of the pyridine group. P4VP is soluble in aqueous media below pH at around 3 because of protonation of its pyridine groups (>96%). At pH around 5 or above, P4VP has either very low or zero charge density, which results in precipitation. P4VP-based polymers have been used for syntheses of polymeric micelles (Koh et al., 2007), microgels (Ma and Fukutomi, 1991; Kim and Vincent, 2005), nanocomposite particles (Fujii et al., 2005, 2006a), surface-modifier for immobilization of nanoparticles (Malynych et al., 2002) and polymer brushes (Wang et al., 2017). Here, P4VP homopolymer was used as a colloidal stabilizer to synthesize PS latex particles.

**Figure 2:**
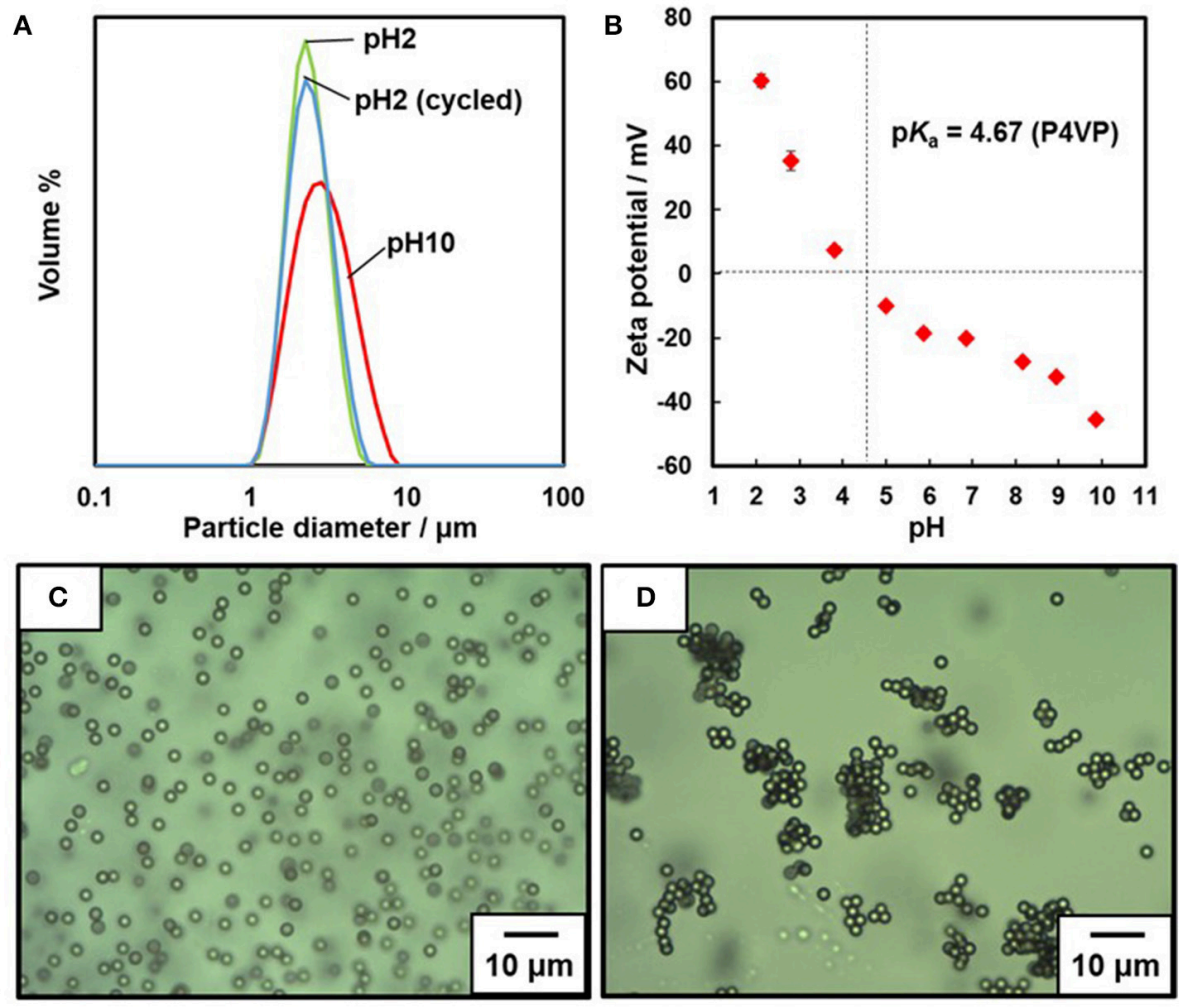
**(A)** Particle size distribution curves measured by laser diffraction method. **(B)** Relationship between pH and zeta potential measured for the P4VP-PS particles. Optical micrographs of aqueous dispersions of P4VP-PS particles observed at **(C)** pH 2.2 and **(D)** pH 10.4.

The number-average molecular weight (*M*_n_) and molecular weight distribution (*M*_w_/*M*_n_) values of the P4VP stabilizer were determined to be 13,200 g/mol and 1.5 by gel-permeation chromatography (GPC). Free radical dispersion polymerization of styrene was carried out using the P4VP colloidal stabilizer, which led to colloidally stable milky dispersion of P4VP-PS particles. SEM studies clarified that the P4VP-PS particles were nearly monodisperse and had a number-average diameter (*D*_n_) of 2.44 ± 0.13 μm (Supplementary Figure 1). Elemental microanalysis indicates a P4VP loading% of 1.05 wt%: we compared the nitrogen content of the P4VP-PS particles (*N* = 0.12%) to that of the P4VP homopolymer (*N* = 11.41%). After replacing the dispersing media from ethanol to distilled water (~pH 6.3), particle size distributions were obtained by the laser diffraction method for dilute dispersion at acidic and basic pHs (Figure 2A). The volume average diameter *D*_v_ was 2.58 ± 0.76 μm at pH 2.1 with a narrow particle size distribution, which indicated that the P4VP-PS particles were well dispersed. The P4VP-PS particles were flocculated at pH 10.4, reflected in an increase in the apparent particle diameter and diameter distribution (*D*_v_, 3.29 ± 1.39 μm). These laser diffraction results agree with observations made by OM (Figures 2C,D): colloidally stable particles at pH 2.2 were detected, whereas a few micrometer to a few tens micrometer-sized flocs were observed at pH 10.4. Adjustment of the solution pH from 10.4 back to 2.3 led to redispersion of the P4VP-PS particles: the *D*_v_ was 2.65 ± 0.85 μm, which is almost the same as that obtained originally at pH 2.1 (Figure 2A). This flocculation/redispersion cycle is reversible at least five times. *D*_v_ determined by the laser diffraction method at pH 10.4 was smaller than *D*_n_ estimated for aqueous dispersion by using OM at pH 10.4. This difference could be caused by breakage of flocs under shear stress during the laser diffraction particle size measurements. (Note that no shear stress was applied during OM observation).

**Figure 3:**
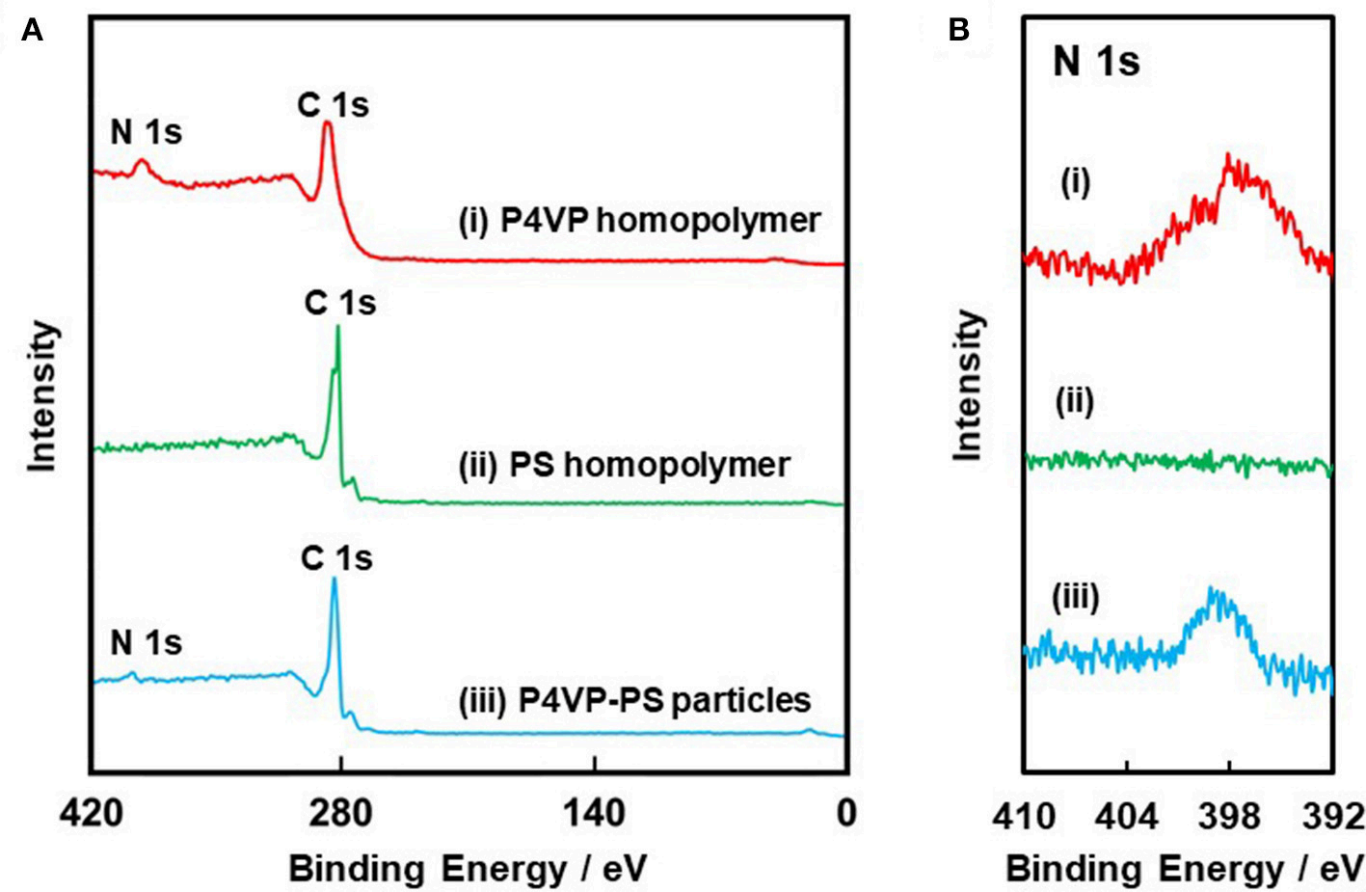
**(A)** XPS survey spectra obtained for (i) P4VP homopolymer, (ii) PS homopolymer, and (iii) P4VP-PS particles. **(B)** Core-level N 1s spectra.

XPS survey spectra and nitrogen core-line spectra obtained for the P4VP-PS particles, the P4VP homopolymer, and PS homopolymer are shown in Figure 3. For both the P4VP-PS particles and the P4VP homopolymer, carbon and nitrogen were detected. On the other hand, nitrogen was not detected, while carbon was observed, for the PS homopolymer. Considering that the XPS can investigate surface chemical compositions with typically ~10 nm, these results verify that the particles are covered by P4VP. Furthermore, a surface coverage by P4VP was determined to be ~27% by comparing the intensity of the N1s signal observed for the P4VP-PS particles to that of the P4VP homopolymer (Table 1). From these XPS and the above-mentioned elemental microanalyses results, it can be confirmed that the P4VP is mainly located at the surface of the PS particles, rather than buried within the PS particles: If the P4VP (1.05 wt% loading on the P4VP-PS particles) existed within the PS particles, nitrogen could not be detected in the XPS spectrum. It is also expected that P4VP exists on the particle surface in aqueous media, because hydrophilicity of the P4VP is higher than that of the PS; Solubility parameters are calculated to be 20.05 (MPa)^1/2^ and 23.31 (MPa)^1/2^ for PS and P4VP (Fedors, 1974). P4VP is expected to be adsorbed to PS particle surface physically in loop-train-tail manner. There is a possibility that the P4VP is chemically grafted *via* chain transfer reaction followed by formation of P4VP-*g*-PS during free radical dispersion polymerization of styrene. The P4VP strongly adsorbed to the PS particles, because the P4VP-PS particles can be dispersed in acidic medium even after five times pH cycles between 2 and 10. Diameter of gyration of the P4VP stabilizer chain was calculated to be 2.5 nm, which was less than the square root of the occupied molecular area (7.45 nm^2^) determined under assumption that the P4VP exists only on the surface of PS particles.

**Table 1:** Quantitative surface composition of PS homopolymer, P4VP homopolymer, and P4VP-PS particles determined by XPS.

	**Atom. %**		**Surface coverage %**	
	**C content/%**	**N content/%**	**P4VP/%**	**PS/%**
PS homopolymer	100	0.0	—	100
P4VP homopolymer	85.6	14.4	100	—
P4VP-PS particles	95.3	4.7	32.5	67.5

The relationship between zeta potential of the P4VP-PS particles and pH is shown in Figure 2B. At and below approximately pH 4, the zeta potentials are positive and have values up to ~+60 mV. The particle surface is positively charged because of protonation of the pyridine groups of the P4VP stabilizer. The zeta potential values were negative at and above ~pH 5 (near the p*K*_a_ value of P4VP), which is due to deprotonation of the P4VP stabilizer. The non-charged neutral P4VP stabilizer collapsed onto the PS surfaces due to dehydration and the electrophoretic mobility of the particles was determined by the hydroxide anion adsorption amount on the particle surfaces (Beattie and Djordjev, 2004; Roger and Cabane, 2012). The same phenomenon has been observed for PS particles carrying PDEA colloidal stabilizer (Fujii et al., 2009; Sekido et al., 2017a). The aqueous electrophoresis studies confirmed that surface charge density on the P4VP-PS particle surfaces can be controlled by pH. (Note that it is not possible to determine real surface charge density, because the zeta potential is an electric potential in the interfacial double layer at the location of the slipping plane). This zeta potential result also indicated that surface hydrophilicity-hydrophobicity balance depends on pH.

Bubble stability depends on the hydrophilicity-hydrophobicity balance of the particle surfaces, in other words, the wettability of the particles at the air-water interface (Ramsden, 1903; Binks and Horozov, 2006; Studart et al., 2006; Fujii and Murakami, 2008; Hunter et al., 2008; Kruglyakov et al., 2011; Stevenson, 2012; Pugh, 2016; Fujii and Nakamura, 2017); therefore, the behavior of bubbles stabilized with these P4VP-PS particles is expected to change at pH values close to the p*K*_a_ of the P4VP stabilizer in a significant manner. To evaluate bubble forming ability and bubble stability, the bubble formation was estimated after shaking aqueous dispersions of the P4VP-PS aqueous latex particles (5.0 wt%) at different pHs. It appears that reasonably stable bubbles, which were stable for at least 4 days, were formed under conditions where the P4VP colloidal stabilizer shows hydrophobic nature and the particles are weakly flocculated in bulk (pH ≥ 4.0). In these cases, the bubbles floated up to planar air-water interface of the aqueous dispersion due to buoyancy. On the other hand, no bubble could be prepared at low pH (e.g., 2.0, 3.0), where the particles have cationic and water soluble P4VP colloidal stabilizer.

**Figure 4:**
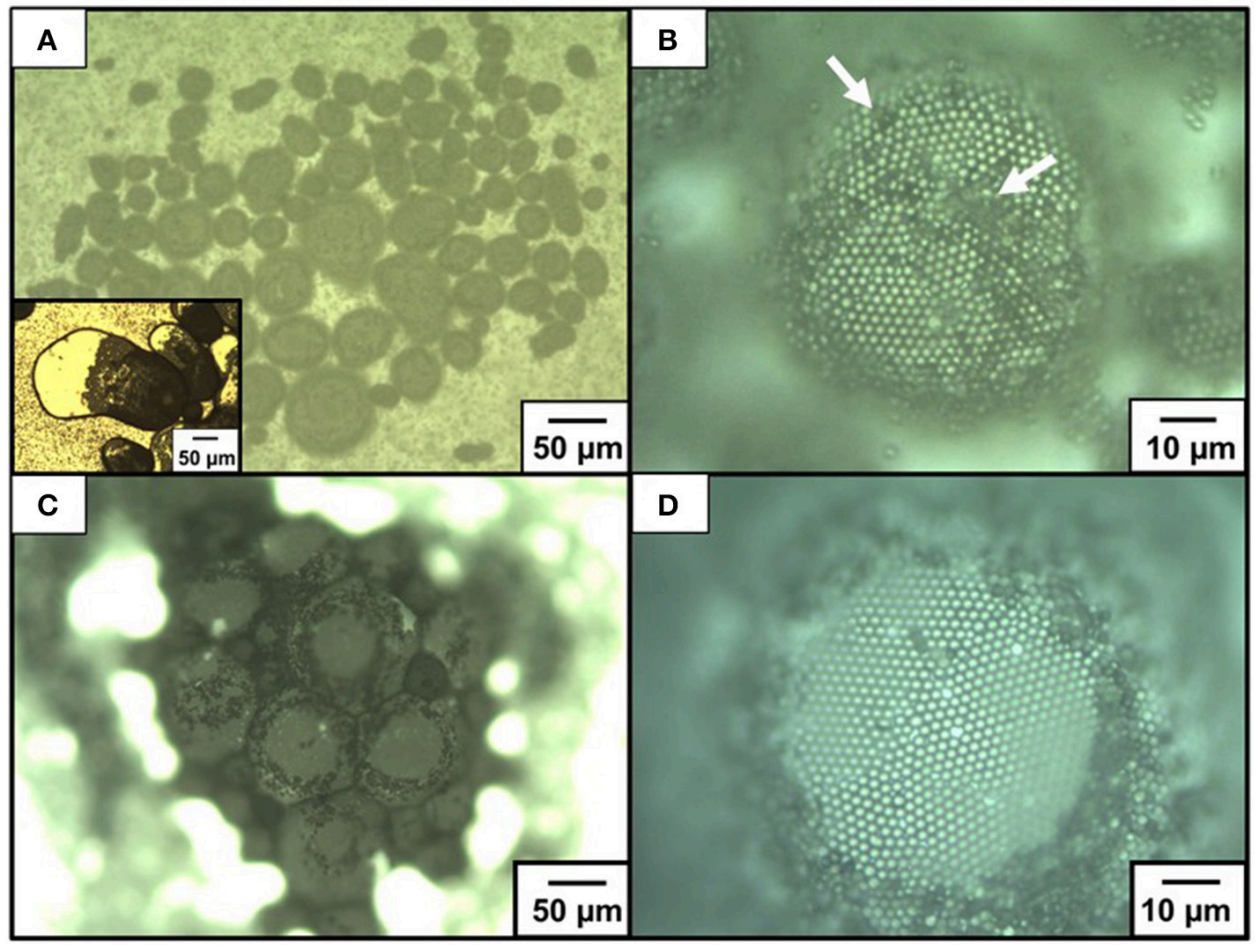
Optical micrographs of bubbles stabilized with P4VP-PS particles at pH 10.4: **(A,B)** before and **(C,D)** after drying. **(B,D)** are magnified images of **(A,C)**, respectively. White arrows indicate flocs of the P4VP-PS particles on bubble surface. An inset of **(A)** shows the bubbles after application of pressure between glass substrates.

**Figure 5:**
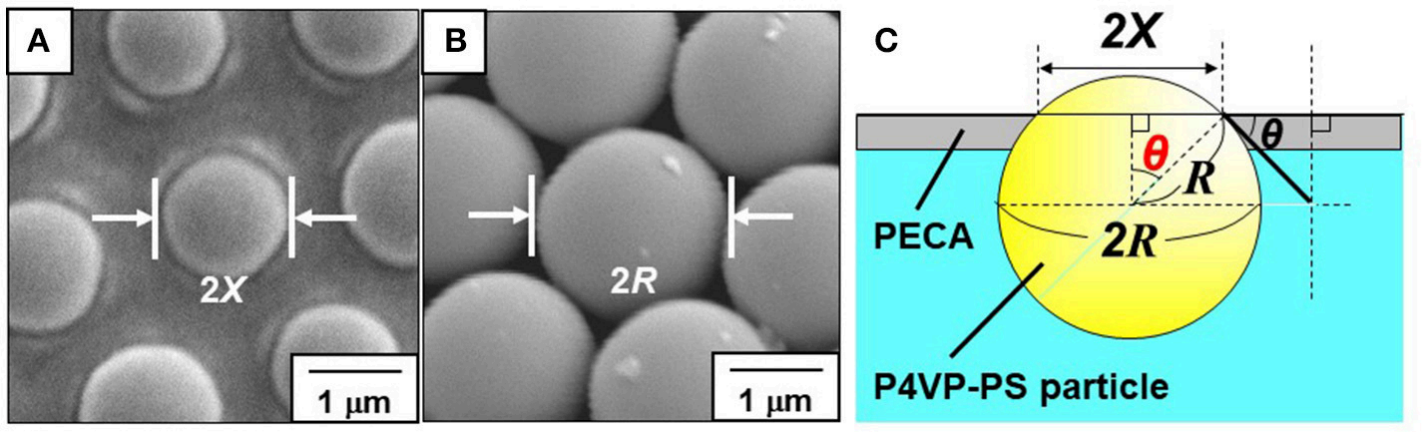
**(A,B)** SEM images of P4VP-PS particles trapped with PECA films recorded from the **(A)** air-exposed and **(B)** water-exposed sides of the films. **(C)** Determination of the contact angle (through water) of the P4VP-PS particle at the air–water interface using SEM images.

**Figure 6:**
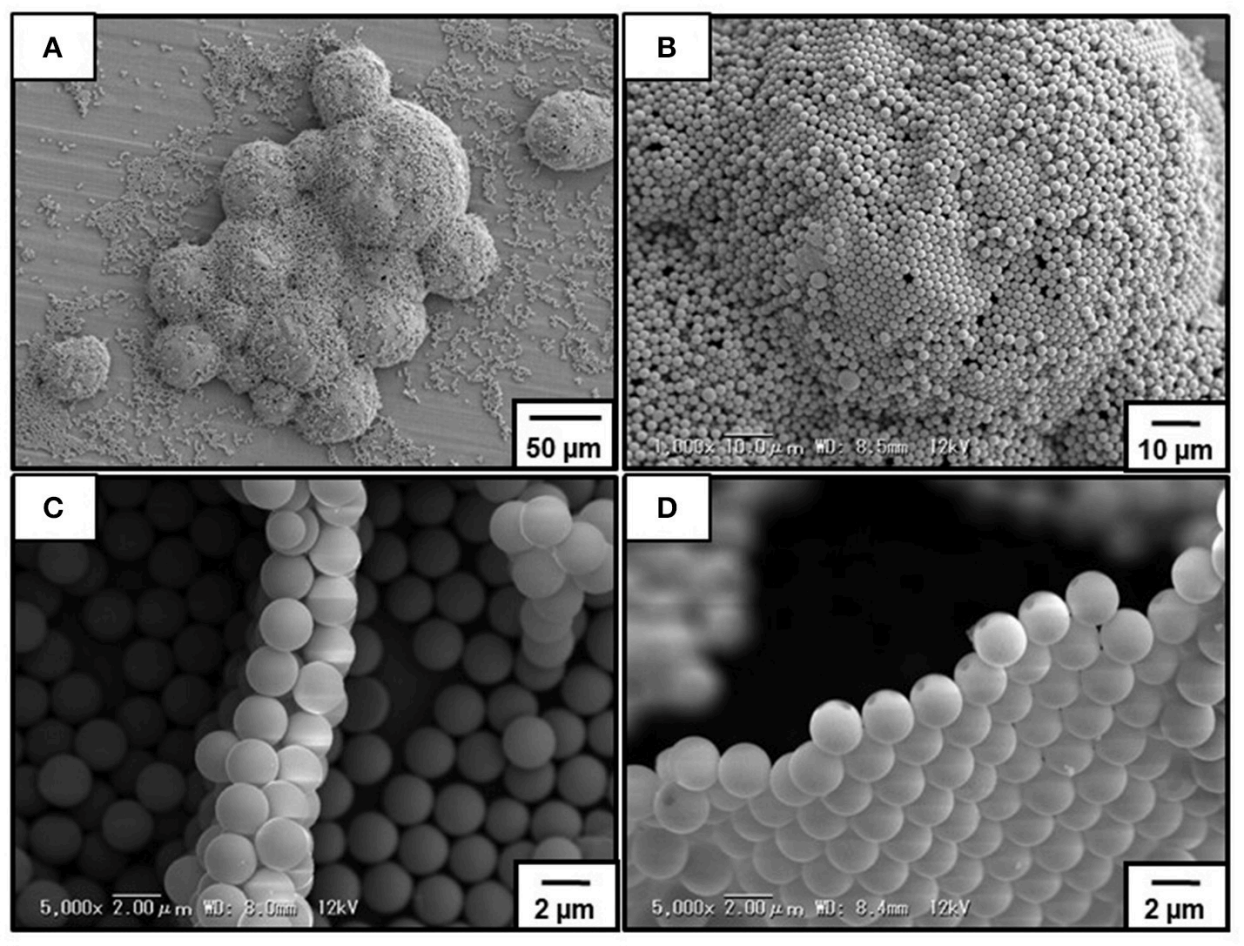
SEM images of dried bubbles stabilized with P4VP-PS particles prepared at pH10.4. **(B)** is a magnified image of **(A)**. **(C,D)** are cross-section SEM images of the bubbles after deliberate rupture using a scalpel.

OM and SEM studies were conducted to investigate the microstructures of the particle-stabilized bubbles (Figures 4–6). An OM study of an aqueous dispersion of the P4VP-PS particles at pH 2.2 after hand shaking, where ~100% of the P4VP colloidal stabilizer is protonated, confirms that the particles do not stabilize the bubbles and are dispersed in the aqueous medium rather than adsorbed at air-water interface. At and above pH 4.0, where less than 76% of the pyridine unit of P4VP colloidal stabilizer is protonated (calculated using Equation 1) and the particles are weakly flocculated in the aqueous media, near spherical and non-spherical, polydisperse bubbles (size range from ~10 μm to ~1 mm) with the P4VP-PS particles adsorbed at the bubble surfaces were observed in the continuous aqueous media (Figure 4A, Supplementary Figures 2, 3). The non-spherical bubbles could be formed by elongation during the hand shaking due to uneven shearing, followed by covering with the P4VP-PS particles before relaxing back to a spherical shape. These non-spherical bubbles might also be formed due to the coalescence of multiple particle-coated bubbles. The bubbles could not become spherical owing to the solid-like properties conferred to the interface by the presence of the P4VP-PS particles (*i.e*., the particles were irreversibly adsorbed at the air-water interface). Similarly, non-spherical bubbles (Subramaniam et al., 2005; Fujii et al., 2018) and oil droplets (Kim et al., 2008; Fujii et al., 2013), which are stabilized with solid particles, have been observed in aqueous media in other studies. Highly magnified OM images of the bubble surface indicated the formation of hexagonal-close-packed arrays of the particles with some flocs on them (Figure 4B) (white arrows indicate the flocs). These particle arrays at air-water interface have been observed in previous studies (Fujii et al., 2006b,c, 2012; Dupin et al., 2008; Nakayama et al., 2015a; Fukuoka et al., 2016; Sekido et al., 2017a,b). It is worth noting that the bubbles were not fully covered with flocculated P4VP-PS particles. There is a possibility that only the P4VP-PS particles adsorbed at air-water interface could remain on the bubble surfaces and flocculated particles detached from the floating bubble surfaces and precipitated in continuous aqueous media. After manual application of light pressure to aqueous gas bubbles placed between a glass slide and a cover glass, the air escaped from the P4VP-PS particle-stabilized bubbles, which indicated encapsulation of air bubble (Figure 4A inset). Magnified image of the crushed bubble indicates that single P4VP-PS particle monolayers were formed at air-water interface to stabilize the bubbles (Supplementary Figure 4). Contact angle θ of the P4VP-PS particles at the air-water interface was evaluated by the interfacial particle trapping method and neglecting gravity (Vogel et al., 2014). The P4VP-PS particles adsorbed at air-water interface was exposed to ethyl 2-cyanoacrylate vapor. Contact of ethyl 2-cyanoacrylate monomer with the interface induced anionic polymerization to form poly(ethyl 2-cyanoacrylate) (PECA) films, which trapped the P4VP-PS particles at the air-water interface. In the SEM images, a spherical cap of each particle was observed on the air phase-exposed side of the film and the spherical particles was observed on the water-exposed side for the film prepared at pH 10.0 (Figures 5A,B). The contact angle θ of the P4VP-PS particles was determined to be 43°, which accorded well with that measured for poly(*N*-vinylpyrolidone)-stabilized PS particle (Fujii et al., 2012), using the diameter of spherical cap of PS particles and the diameter of original P4VP-PS particles (Figure 5C). The adsorption energy of the P4VP-PS particle adsorbed at an air-water interface from the liquid phase (Δ*G*) can be calculated to be 4.1 × 10^6^ *k*_B_*T* with the contact angle using Equation (2) (Levine et al., 1989):

(2) $$\begin{array}{r} \Delta G=-\gamma_{aw}\pi a^{2}{\left(1-\cos\theta \right)}^{2} \end{array}$$

where *k*_B_ is the Boltzmann constant and *T* is temperature, γ_aw_ is the surface tension of water, *a* is the particle radius, and θ is the contact angle measured through the aqueous phase.

After evaporation of the continuous water phase from the aqueous bubbles prepared at pH 10.4 overnight at ambient temperature, solid bubbles with three-dimensional structures were obtained (Figure 4C). A little coalescence was observed and the bubble size increased during/after drying, which was confirmed by OM studies. After the water evaporation, near-spherical bubble shapes observed when dispersed in water medium were significantly deformed. Due to capillary forces working among the particle-stabilized bubbles during drying, the bubbles were forced to deviate from their near-spherical shape. Because of the deformability of the P4VP-PS particle layer formed at bubble surface, the bubble deformation could not be avoided during/after drying. It is worth noting that the particle arrays remained even after drying (Figure 4D).

Figure 6 shows SEM images obtained for dried bubbles prepared at pH 10.4. Even under high vaccuum condition, the bubbles kept their three-dimensional structure (Figure 6A). Observation of the top surface of the dried bubbles indicated the presence of the P4VP-PS particles, which were near close-packed (Figure 6B). The internal particle microstructure was examined after deliberate rupture of the bubbles using a scalpel. Well-defined particle bilayers were observed in most cases (Figure 6C), which strongly indicates that most bubbles were stabilized by P4VP-PS particle monolayers. During water drainage from the drying foams, these monolayers should be forced together to form bilayers. Similar results were reported previously (Fujii et al., 2006b,c; Dupin et al., 2008; Nakayama et al., 2015a; Fukuoka et al., 2016). In some cases, monolayers and multilayers (mainly triple layers) were observed. The monolayers should be due to the top surface of dried bubbles that contacted with bulk air phase in direct manner and was not overlapped with other bubbles (Figure 6D). The multilayers should be formed due to flocs on the bubble surfaces and/or excess free particles trapped between two bubble surfaces.

**Figure 7:**
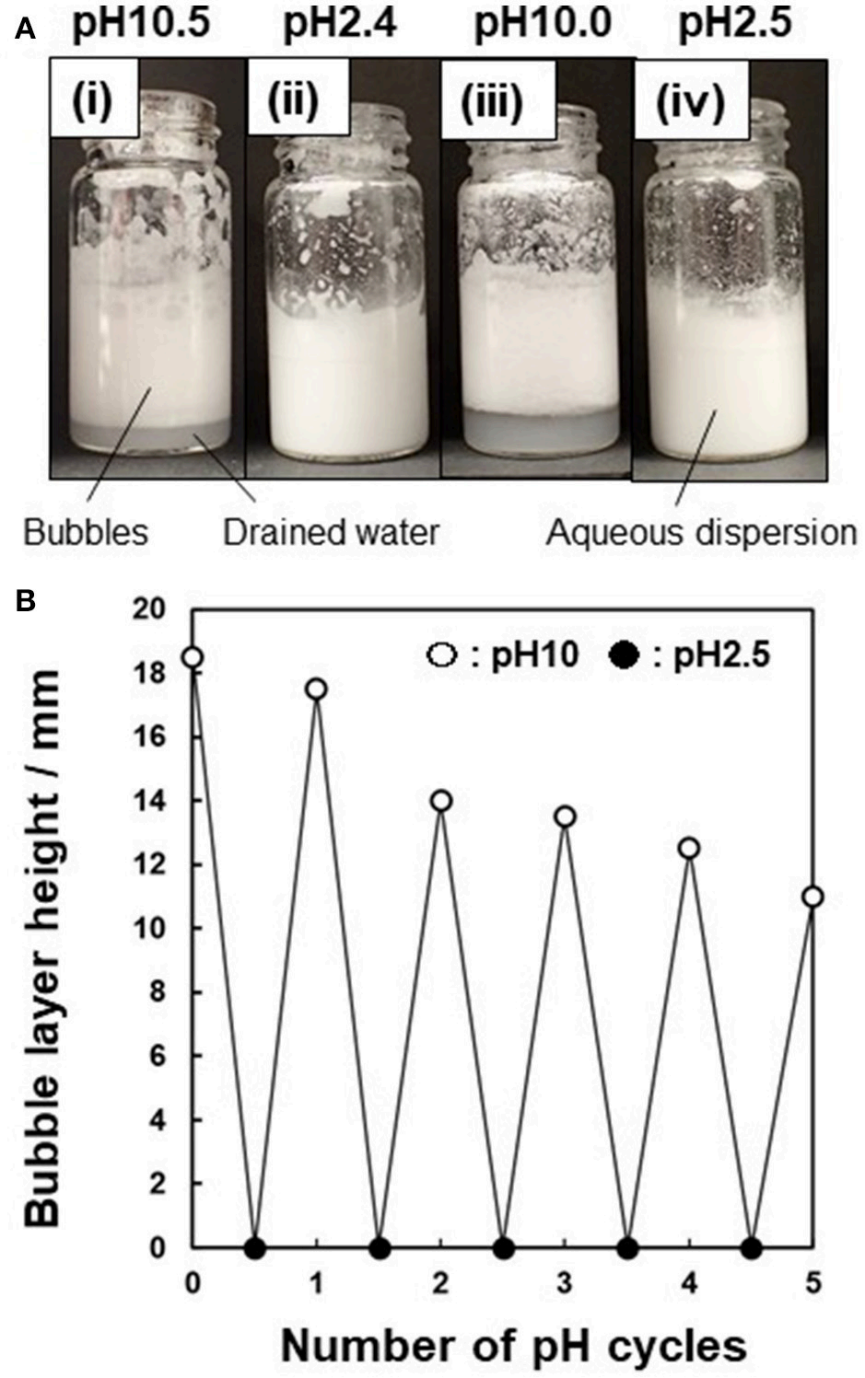
**(A)** Photographs illustrating pH-responsive behavior of bubbles prepared using P4VP-PS particles (5.0 wt %): (i) at pH 10.5 after 10 min, (ii) pH adjustment from pH 10.5 to pH 2.4 after 10 min, (iii) pH adjustment from pH 2.4 to pH 10.0 after 10 min, and (iv) pH adjustment from pH 2.4 to pH 10.0 to pH 2.5 after 10 min. **(B)** Height of the bubbles layer vs. the number of pH cycles.

Finally, the possibility of inducing destabilization of the particle-stabilized bubbles by subsequent pH control was investigated. The bubbles formed at pH 9.9 and allowed to stand for 10 min after preparation were rapidly (< 1 min) destabilized (coalesced) by decreasing the pH of the aqueous phase to pH 2.2, followed by vigorous hand shaking at 25°C. This should be due to *in situ* protonation of the P4VP colloidal stabilizer on the P4VP-PS particles, which rendered the particle surface highly hydrophilic. Therefore, the P4VP-PS particles are detached from the air-water interface and are not adsorbed at the interface anymore, which leads to disruption of the bubbles. The bubbles could be reformed after pH adjustment back to pH 10 followed by hand shaking. This stabilization/destabilization cycle is readily reversible at least five times (Figure 7).

## Conclusion

In summary, near-monodispersed, micrometer-sized PS particles carrying P4VP colloidal stabilizer on their surfaces were successfully synthesized by free radical dispersion polymerization. The particle were characterized in terms of the morphology, size, size distribution, chemical composition, surface chemistry, and pH-response. The ability of the P4VP-PS particles was evaluated as a pH-dependent and pH-responsive particulate bubble stabilizer. Aqueous bubbles can be stabilized with the P4VP-PS particles at and above pH 4.0, where the particles have relatively hydrophobic surfaces. On the other hand, no bubble was stabilized at and below pH 3.0, where the particles are positively charged and colloidally stable. Destabilization of the bubbles prepared at pH ~10 could be induced by subsequent pH adjustment to ≤ 3.0, and the stabilization/destabilization cycles were reversible. Recently, it has been confirmed that there are lots of similarities among particle-stabilized bubbles, emulsions and liquid marbles/dry liquids (Fujii et al., 2016). The principles demonstrated in this study should also be applicable to predict the stabilities and microstructures of these particle-stabilized soft dispersed systems. The encapsulation of air bubbles in liquid phase using solid particles with stimuli-responsive character should be useful in food manufacturing, personal care products and cosmetic formulations.

## Author contributions

MI, KT, HH, YA, and SY carried out the experiments with respect to synthesis and characterization of the P4VP-PS particles and bubble formation and characterization experiments. SF organized the project and wrote the manuscript. All authors discussed the results and edited the manuscript. YA conducted additional experiments (XPS studies and contact angle measurement of the P4VP-PS particle at air-water interface) and discussed the results.

### Conflict of interest statement

The authors declare that the research was conducted in the absence of any commercial or financial relationships that could be construed as a potential conflict of interest. The reviewer, YW, and handling Editor declared their shared affiliation.
