# Supplementary material for: pH-Responsive Aqueous Bubbles Stabilized With Polymer Particles Carrying Poly(4-vinylpyridine) Colloidal Stabilizer
Source: Front Chem. 2018 Jul 17;6:269. doi: 10.3389/fchem.2018.00269 (PMC6056661; doi:10.3389/fchem.2018.00269)
Supplement: Supplementary file 1 [file Data_Sheet_1.pdf]

Supplementary Material

pH-Responsive aqueous bubbles stabilized with polymer particles carrying poly(4-vinylpyridine) colloidal stabilizer

Masaya Ito, Koki Takano, Haruka Hanochi, Shin-ichi Yusa, Yoshinobu Nakamura, Syuji Fujii*

*** Correspondence:** Prof. Syuji FUJII: syuji.fujii@oit.ac.jp

Gel-permeation chromatography (GPC)

GPC measurement was carried out for P4VP homopolymer with a Jasco UV-2075 detector equipped with a Shodex OHpak SB-804 HQ column working at 40 ˚C with a flow rate of 0.6 mL/min. An acetic acid (0.5 M) solution containing sodium sulfate (0.3 M) was used as the eluent. Number-average molecular weight (M_n) and molecular weight distribution (M_w/M_n) were calibrated using standard poly(2-vinylpyridine) samples.

Digital camera

Optical photographs of the samples were taken using a digital camera (GX200, Ricoh).

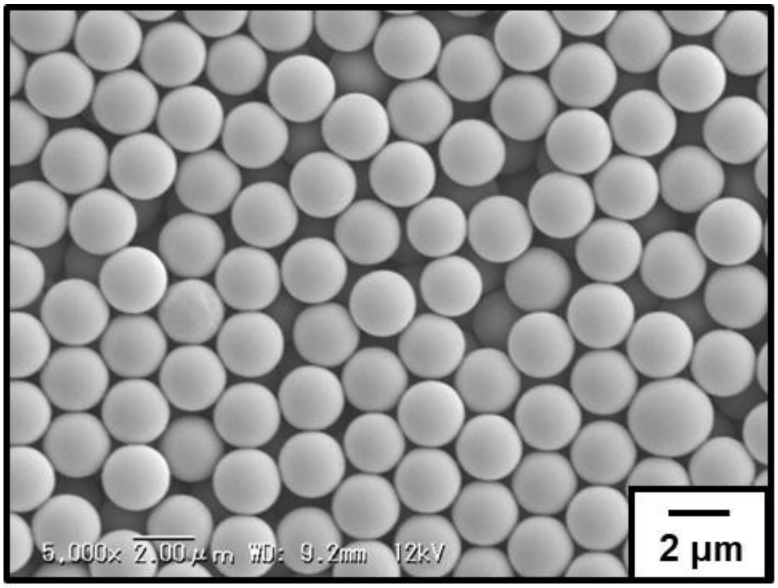

Supplementary Figure 1. SEM image of the P4VP-PS particles used in this study.

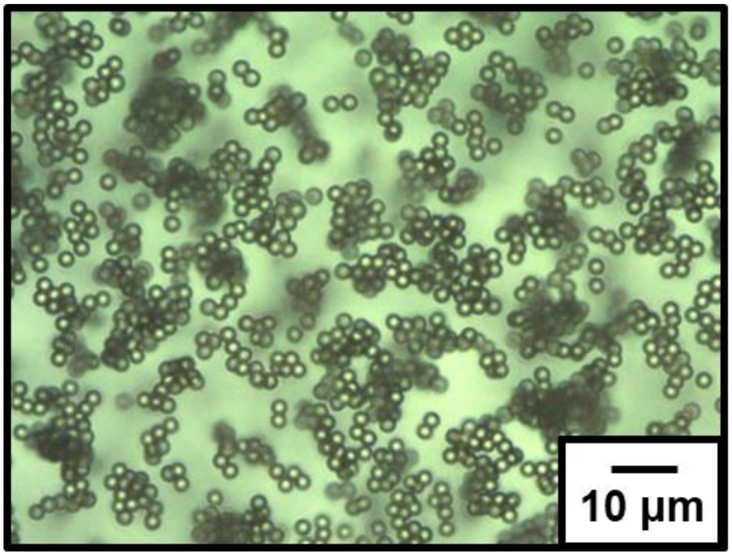

Supplementary Figure 2. Optical micrograph of aqueous dispersion of P4VP-PS particles observed at pH 4.0.

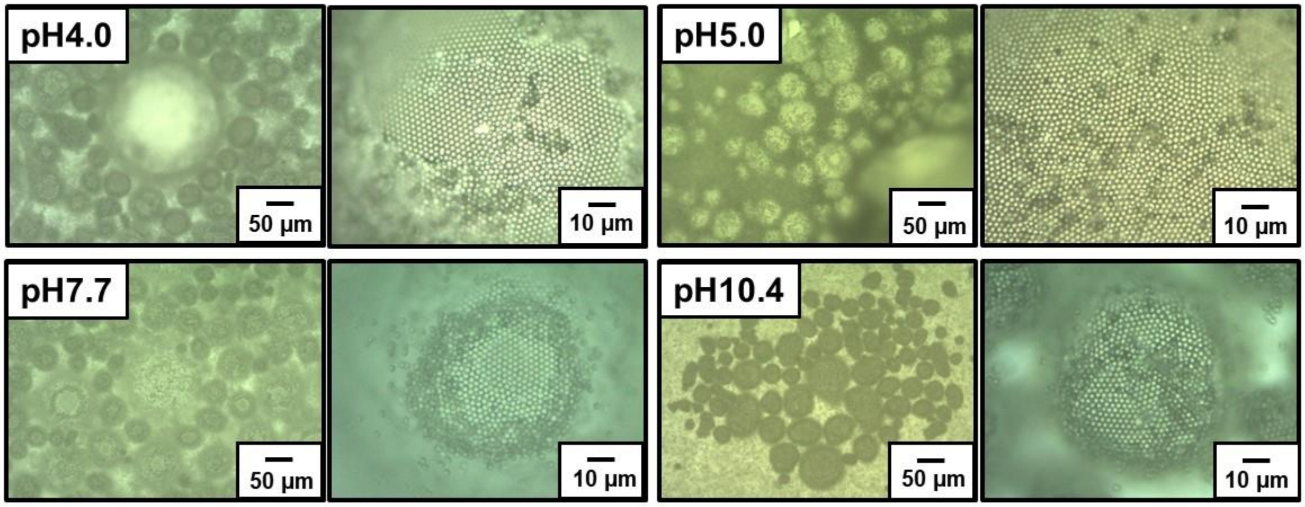

Supplementary Figure 3. Optical micrographs of bubbles stabilized with P4VP-PS particles at pH 4.0, 5.0, 7.7 and 10. Right side images are magnified images of left side images.

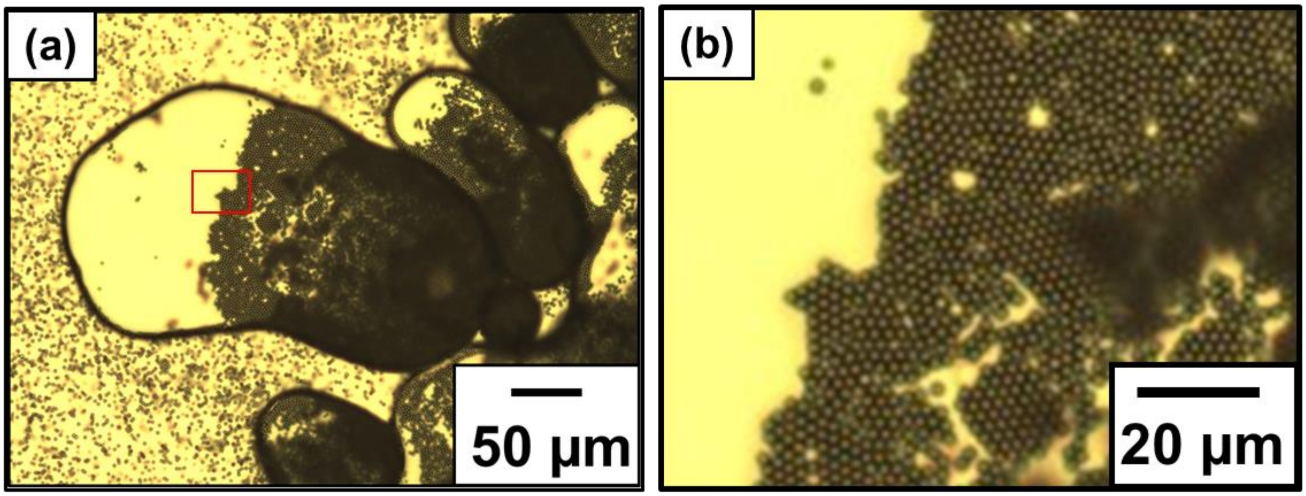

Supplementary Figure 4. (a) Optical micrograph of a crushed air bubble. (b) Magnified image of the area shown in (a).
